# PC945, a Novel Inhaled Antifungal Agent, for the Treatment of Respiratory Fungal Infections

**DOI:** 10.3390/jof6040373

**Published:** 2020-12-17

**Authors:** Alison Murray, Lindsey Cass, Kazuhiro Ito, Nicole Pagani, Darius Armstrong-James, Paras Dalal, Anna Reed, Pete Strong

**Affiliations:** 1 Pulmocide Ltd., Office Suite 3.01, 44 Southampton Buildings, London WC2A 1AP, UK; Alison@pulmocide.com (A.M.); Lindsey@pulmocide.com (L.C.); Kaz@pulmocide.com (K.I.); 2 Royal Brompton and Harefield NHS Foundation Trust, Respiratory and Transplant Medicine, Harefield Hospital, Harefield UB9 6JH, UK; nicole.pagani@nhs.net (N.P.); P.Dalal@rbht.nhs.uk (P.D.); A.Reed@rbht.nhs.uk (A.R.); 3 Department of Infectious Diseases, Imperial College London, London SW7 2AZ, UK; d.armstrong@imperial.ac.uk

**Keywords:** *Aspergillus fumigatus*, triazole, inhalation

## Abstract

Disease due to pulmonary *Aspergillus* infection remains a significant unmet need, particularly in immunocompromised patients, patients in critical care and those with underlying chronic lung diseases. To date, treatment using inhaled antifungal agents has been limited to repurposing available systemic medicines. PC945 is a novel triazole antifungal agent, a potent inhibitor of CYP51, purpose-designed to be administered via inhalation for high local lung concentrations and limited systemic exposure. In preclinical testing, PC945 is potent versus *Aspergillus* spp. and *Candida* spp. and showed two remarkable properties in preclinical studies, in vitro and in vivo. The antifungal effects against *Aspergillus fumigatus* accumulate on repeat dosing and improved efficacy has been demonstrated when PC945 is dosed in combination with systemic anti-fungal agents of multiple classes. Resistance to PC945 has been induced in *Aspergillus fumigatus* in vitro, resulting in a strain which remained susceptible to other antifungal triazoles. In healthy volunteers and asthmatics, nebulised PC945 was well tolerated, with limited systemic exposure and an apparently long lung residency time. In two lung transplant patients, PC945 treated an invasive pulmonary *Aspergillus* infection that had been unresponsive to multiple antifungal agents (systemic ± inhaled) without systemic side effects or detected drug–drug interactions.

## 1. Introduction

Fungi are an important cause of lung disease, as fungal spores are ubiquitous in the environment and the small size of spores of species such as *Aspergillus* facilitates inhalation and deposition in the distal airways [1,2]. In patients with impaired mucociliary and/or macrophage/neutrophil function, retained mucous or compromised airways, inhaled spores can persist and spread within the airways [3]. The temperature and moist environment in the airways provide ideal conditions for germination and the establishment of infection particularly *Aspergillus* spp. [3]. Where immunity is compromised, tissue invasion and dissemination can occur and in other settings fungal products produced by the fungus such as proteases and gliotoxin cause airway inflammation and remodelling, fibrosis, smooth muscle changes and cell wall derivatives can act as allergens [4,5,6,7].

Fungi both cause and complicate lung disease. Despite the use of antifungal prophylaxis, morbidity and mortality due to invasive aspergillosis remains significant among immune-suppressed patients [8,9,10] and in critically ill patients requiring admission to intensive care post-influenza or post-coronavirus (SARS-COV-2: COVID-19) infections, or following high dose steroids for chronic obstructive pulmonary disease (COPD) [10,11,12,13]. Invasive pulmonary aspergillosis (IPA) accounts for 74–78% of invasive aspergillosis (IA) [14]. *Aspergillus* colonization or infection occurs post-lung transplant in approximately 30–50% of patients [15,16], with IPA being reported in 7.5% of patients [11] and is also associated with an increased risk of chronic lung allograft dysfunction (CLAD). Chronic pulmonary aspergillosis (CPA) complicates numerous respiratory conditions, particularly those characterised by fibrosis or lung cavitation [17,18]. As the burden of pulmonary fibrosis after COVID-19 recovery could be substantial [19,20], the prevalence of CPA is likely to increase.

Fungi also play a significant role in allergic airways disease exemplified by allergic bronchopulmonary aspergillosis (ABPA), which is a well-described but relatively uncommon endotype of severe asthma; up to 70% of patients with severe asthma exhibit sensitization to different fungi [21,22,23]. In general, patients are treated with inhaled steroids and biologics (such as anti-IgE, anti-IL-5 antibody) focusing on the allergic response. Work suggests that the key allergen is a product of active metabolism, a protease [4,6,24]. If this is so, then live fungus in the airways would be the underlying cause of the immuno-inflammatory response and therefore amenable to treatment with an appropriate antifungal agent [25,26].

## 2. Limitations of Current Antifungal Agents

Currently available antifungal therapies have important limitations, including route of administration or dosing forms, treatment-limiting side effects and drug–drug interactions (DDIs) [10,16]. DDIs are a significant challenge with the azoles, particularly with specific classes of concomitant drugs in some at-risk patient groups, such as calcineurin inhibitors in lung transplant recipients, vincristine in patients undergoing treatment for acute lymphoblastic/lymphocytic/lymphoid leukemia (ALL) [27] and checkpoint inhibitors in patients being treated for acute myeloid leukemia (AML). Corticosteroids are also a standard therapy for ABPA but there are now numerous reports of toxicity using corticosteroid in combination with itraconazole resulting in adrenal suppression in both asthma and cystic fibrosis (CF) patients with ABPA, owing to itraconazole inhibition of the hepatic cytochrome P450 3A4, which is responsible for metabolic clearance of corticosteroid [28,29].

Many approved antifungal agents have a narrow therapeutic window. Dose reductions required to address DDIs exacerbate this problem in many at-risk patient groups. Hepatic and cardiac toxicity are common with the azoles, and discontinuation rates are high resulting in curtailment of treatment in a large proportion of patients [30]. Systemic amphotericin B is used with caution in groups, such as lung transplant recipients, due to the risk of nephrotoxicity. Although DDIs occur less frequently with echinocandins, these are not recommended as monotherapy for the primary treatment of IPA given their relatively modest inherent antifungal effects [31]. Delivery via the parenteral route largely restricts the use of echinocandins and amphotericin B to short-term use in inpatient settings. In lung transplant recipients the anastomotic site is particularly vulnerable to aspergillus infection due to the presence of the sutures that act as a point of adhesion. This, together with the disruption of blood supply to the donor airway wall and the resulting post-transplantation ischaemia, limits delivery of systemic antifungal agents to the site of infection.

As a result of these limitations, current rates of response to treatment of IA are low (≤50%) and pulmonary fungal infection remains an important cause of morbidity. Attributable mortality remains high, ranging from 14 to 40%, even when treatment includes the most recently approved systemic products [32,33,34].

There is, therefore, a critical unmet need for improved antifungal agents for the treatment of pulmonary aspergillus disease. A potent, effective inhaled anti-fungal agent with prolonged lung tissue residence would be a valuable adjunct to current therapeutic options. The idea of delivering an anti-fungal agent directly to the airway with limited systemic uptake is intuitively appealing and has the goal of delivering a high concentration of drug to the infected area while avoiding systemic toxicity [16]. An inhaled approach is a particularly attractive option post-lung transplant for delivering drug to the anastomotic site, which is poorly vascularised and, hence, difficult to access via a parenteral route of administration. However, there are no approved inhaled antifungal therapies. Safety and tolerability are poor as nebulization causes airway irritation, coughing, nausea, and vomiting. Patients find it particularly unpleasant to inhale due to its smell and taste [16,31]. As a result, compliance with nebulised amphotericin B therapy is poor. Hilberg and colleagues presented three cases of IPA treated with inhaled voriconazole solutions. It was found to be safer and showed better therapeutic effectiveness in the subjects compared with oral administration, but it was absorbed quickly, as reflected by systemic concentrations, and is not optimised as an inhaled medicine since it is designed to be rapidly and extensively absorbed from the gut and so is unlikely to provide sustained exposure in the lung and airways [35,36]. Recently, inhaled forms of itraconazole (PUR1900 (Pulmatrix Inc.)) and voriconazole (ZP059 (Zambon group), TFF-VORI (TFF Pharmaceuticals)) have entered clinical trials [37,38,39], but they are for repurposed use, and no compound designed specifically, and optimised for inhalation therapy, is currently available.

Therefore, there is still a significant unmet need for an effective inhaled, antifungal agent that patients will find easy to tolerate, cause fewer systemic side effects and DDIs than current antifungal agents, and that can be used in both in- and outpatient settings to treat invasive *Aspergillus* infections, including those at the anastomotic site following lung transplantation. The safety profile of such an agent, coupled with its efficacy, would provide the opportunity to investigate the role of *Aspergillus* in patients with problematic chronic respiratory diseases, including ABPA, severe asthma and CPA.

## 3. PC945: Preclinical Profile

**Figure 1:**
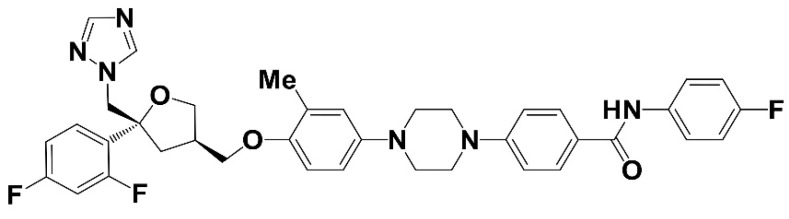
Chemical Structure of PC945.

PC945, which has the chemical formula 4-[4-(4-{[(3*R*,5*R*)-5-(2,4-difluorophenyl)-5-(1*H*-1,2,4-triazol-1-ylmethyl)oxolan-3-yl]methoxy}-3-methylphenyl)piperazin-1-yl]-*N*-(4-fluorophenyl)benzamide (Figure 1) is a novel triazole which has been optimised for inhaled delivery [40], with minimal systemic exposure. It potentially addresses at least some of the unmet needs discussed above by maximising the potential for therapeutic activity in the lungs while minimising the potential for toxicity in other organs.

In vitro, as for other triazole anti-fungal compounds, PC945 was shown to be a tightly binding inhibitor of *A. fumigatus* sterol 14-demethylase (CYP51A and CYP51B) and strongly inhibited ergosterol synthesis in *A. fumigatus* with an IC_50_ of 6.9 nM, which was 14- and 2.6-fold more potent than voriconazole and posaconazole, respectively [41]. Thus, the primary mechanism of PC945 is depletion of ergosterol in the fungal membrane, which disrupts the structure and many membrane functions leading to inhibition of fungal growth.

Against 96 clinically isolated *A. fumigatus* strains obtained in France and the United Kingdom, using a EUCAST method, the geometric mean MIC of PC945 was 0.17 μg/mL, and the MIC_50_ and MIC_90_ values were 0.125 and 1.0 μg/mL, respectively, as shown in Table 1 below [41]. The potency of PC945 was superior to that of voriconazole and comparable to that of posaconazole. The effects of treating human bronchial cells with PC945 were investigated, since inhaled PC945 will be delivered efficiently to the respiratory epithelium. Furthermore, 30–50 percent of adherent *A. fumigatus* conidia are thought to internalise into bronchial epithelial cells, or alveolar cells, as well as producing hyphal growth parallel to the epithelium in vitro [42,43,44]. PC945 was found to be absorbed quickly into cells and to produce persistent antifungal effects [41].

PC945 was found to be effective against a broad spectrum of pathogenic fungi, including *Aspergillus* spp. *Candida* spp., *Trichophyton rubrum*, *Cryptococcus gattii*, *C. neoformans*, *Penicillium chrysogenum* and *Rhizopus oryzae* [41,45] (and unpublished data). With the emergence of *Candida auris* as a significant new healthcare problem, the effects of PC945 versus a total of 72 strains were evaluated. Of those, 53 isolates showed the reduced susceptibility to fluconazole (≥64 mg/L), indicating many strains were fluconazole resistant. PC945 (geometric mean 24 h CLSI MIC, 0.058 mg/L) was 7.4- and 1.5-fold more susceptible than voriconazole and posaconazole, respectively, and the differences were statistically significant [45].

In an in vitro forced mutation induction study, PC945 exhibited a significantly higher barrier to induction of a resistance of *A. fumigatus* compared with itraconazole. In addition, the induced resistance apparently specific to PC945, in that it did not show cross-resistance to other known azoles (itraconazole, posaconazole and voriconazole) [46]. The mechanism of resistance induced to PC945 is presently unknown.

**Figure 2:**
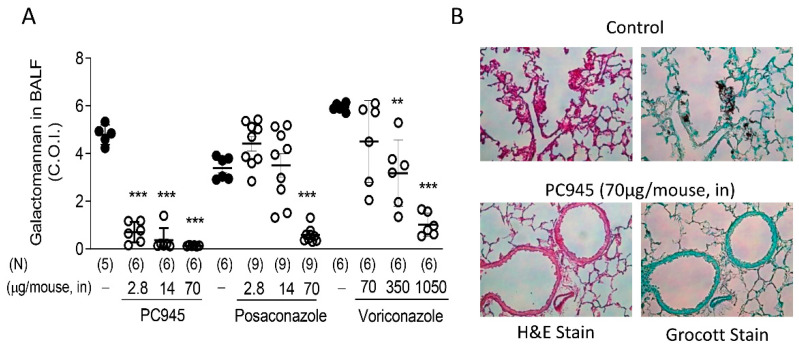
Effects of intranasally dosed PC945, posaconazole or voriconazole in temporarily neutropenic mice infected with *A. fumigatus*. Compounds were dosed on day 0 (30 min before infection) and on days 1, 2, and 3 post infection, and lungs and BALF were collected 6 h after the final dose. Shown are galactomannan levels in BALF (**A**), and haematoxylin-eosin (H&E) staining and Grocott fungus staining (magnification, ×200) of lung sections (**B**). In controls 3 days after *Aspergillus* infection (upper panels) the following features were noted: abundant hyphae growing in alveoli, infiltrating the lung parenchyma; parenchymal destruction and necrosis; scarce inflammatory cell recruitment (cellular inflammatory responses were primarily foamy and activated macrophages, lymphocytes with a few neutrophils). By contrast, in PC945-treated lungs (lower panels) the following features were noted: few or no fungal hyphae; no major disruption of alveoli; parenchymal structure preserved; limited number of foamy macrophages. 1 control animal in PC945 experiment was dropped out before sample collection on day 3 post infection. 3 control mice in posaconazole experiment were taken for preliminary histology analysis. ** *p* < 0.01, *** *p* < 0.001 vs. each infection control. Adapted from [47].

The effects of intranasally dosed PC945 on *A. fumigatus* infections and associated biomarkers have been studied in temporarily neutropenic mice, both as a treatment for an existing infection and as prophylaxis [41,47]. PC945 was significantly more potent than posaconazole or voriconazole when the treatments were given via intranasal dosing (Figure 2A). Particularly, voriconazole required a much higher dose due to its physicochemical properties; voriconazole is not retained in the lung after inhalation and rapidly enters the systemic circulation as demonstrated in patients after voriconazole inhalation [36]. Histology slides from these studies (Figure 2B) show alveolar changes in control animals 3 days after infection with *A. fumigatus*, which are largely prevented by treatment with PC945. Intranasal dosing was used to achieve lung exposure to PC945 in these studies, as the instilled dose was aerosolised on inhalation. Overall, the other main findings of these studies were that:

- PC945 inhibited *Aspergillus* infection (fungal load, serum and BAL galactomannan (GM)) and biomarkers whether treatment was administered prophylactically, immediately before infection or started 24 h post infection.
- The pattern of the effects seen on the different prophylactic regimens explored strongly suggested that antifungal effects of PC945 accumulated in the lungs of mice on repeat dosing. These results clearly indicated that investigation of the PK:PD relationship would be valuable.

**Figure 3:**
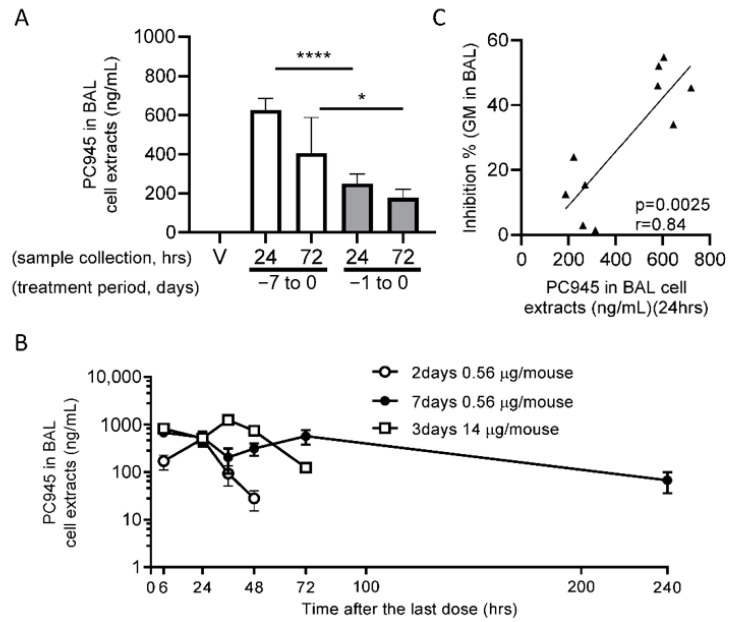
PK:PD relationship for PC945 in *Aspergillus*-infected, temporarily neutropenic mice. PC945 (0.56 µg/mouse) was administered once daily intranasally from day 7 to day 0 or from day 1 to day 0 before *A. fumigatus* inoculation, and BAL was collected 24 h or 72 h after the last dose and *A. fumigatus* inoculation. BAL cells and BAL supernatant were separated by centrifugation. The levels of PC945 in BAL cell extracts are shown in panel (**A**). (**B**) Non-infected intact mice were given PC945 (0.56 µg/mouse) once daily for 7 days or for 2 days, or PC945 (14 µg/mouse) for 3 days, and PC945 concentration in BAL cell extracts was measured at 6, 24, 48, 72 and/or 240 h post the last dose. (**C**) The relationship between PC945 in BAL cell extracts and inhibition of galactomannan (GM) in BAL in *Aspergillus*-infected, temporarily neutropenic mice (E). Each horizontal bar is presented as mean ± SD. 5 mice per group. * *p* < 0.05, **** *p* < 0.0001 by ANOVA-unpaired *t*-test with Welch’s correction. V: vehicle. Adapted from [48].

Figure 3A shows that PC945 was readily detectable among BAL cell pellets in *A. fumigatus* infected mice and Figure 3B shows the time course of PC945 concentrations in non-infected mice [48]. When the relationship between antifungal activity and BAL cell pellet concentrations were investigated, a statistically significant correlation was found between the severity of infection (as judged by BAL GM concentrations) and the concentration of PC945 in BAL cell pellets (Figure 3C). Not only does this suggest that the intracellular compartment is important for understanding the lung PK of PC945, it is interesting that granulocytes or macrophages loaded with anti-fungal triazoles have been suggested to demonstrate enhanced antifungal activity against *A. fumigatus* or *Blastomyses dermatitidis* than naïve cells [49,50]. The superior effects of therapeutic and prophylactic intranasal PC945 observed above for *A. fumigatus*, were also confirmed in *C. albicans* lung infection model in vivo [51].

Since we believed that inhaled treatment with PC945 would be likely to be combined with systemic treatment with another antifungal agent when used to treat invasive disease, the effects of combination therapy versus monotherapy were investigated. We found that combining topical treatment using PC945, with systemic treatment using known triazoles, demonstrated superior antifungal effects against *A. fumigatus* in an in vitro human alveolus bilayer model and in the lungs of neutropenic immunocompromised mice than systemic triazole alone [52].

**Figure 4:**
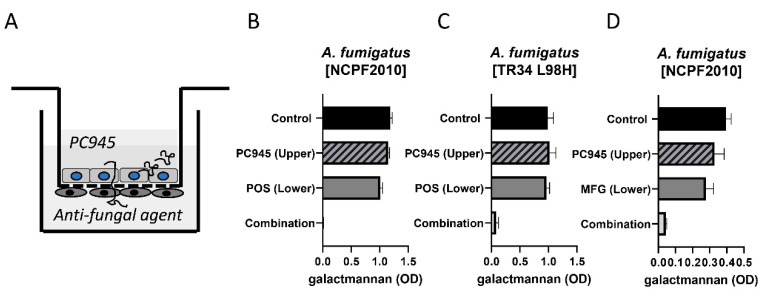
Pharmacodynamic responses to PC945 in an in vitro model of the human alveolus. (**A**) An illustration of the 2-cell model, showing combined treatment with PC945 in the upper (apical chamber and posaconazole in the lower (basolateral chamber. (**B**) effects of treatment with PC945 (0.1 µg/mL) or posaconazole (0.01 µg/mL) alone or in combination versus azole-susceptible fungus on Day 2 post infection; (**C**) effects of treatment with PC945 (0.1 µg/mL) or posaconazole (0.1 µg/mL) alone or in combination versus azole-resistant fungus on day 2 post infection; (**D**) effects of treatment with PC945 (0.1 µg/mL) or micafungin (1 µg/mL) alone or in combination versus azole-susceptible fungus on day 2 post infection. POS: posaconazole, MFG: Micafungin. Adapted from [52,53].

Figure 4 shows results for combined treatment of PC945 with either posaconazole or micafungin versus an azole-susceptible strain or PC945 versus posaconazole versus an azole-resistant strain. Synergy is apparent in all three experiments, and was also confirmed with other antifungal agents (voriconazole, itraconazole, caspofungin and micafungin) [52,53]. Surprisingly, there was little or no synergistic interaction observed when apical and basolateral posaconazole or voriconazole were combined. Echoing the in vivo studies summarised above, repeated prophylactic treatment with PC945 showed superior effects to a single prophylactic dose, suggesting tissue retention and/or accumulation of PC945. In the immunocompromised mouse model discussed above, treatment with a combination of intranasal PC945 and oral posaconazole again showed synergistic benefit (here on animal survival) versus monotherapy with either agent.

The studies undertaken which support the investigation of PC945 in clinical studies are summarized in Table 2. In common with other antifungal agents containing a triazole moiety, PC945 shows inhibition of CYP3A4/5 in human liver microsomal preparations in vitro. After 30 min pre-incubation, the measured PC945 IC_50_ values on CYP3A4/5 were 247 nM and 17 nM for substrates testosterone and midazolam, respectively. There was no inhibitory activity for the other CYP isoforms, however evaluated on a range of CYP450 isoforms using pooled human liver microsomes (personal communication).

Safety pharmacology: PC945 has no significant effects on the general behaviour, physiological state, body temperature or spontaneous locomotor activity in rats. PC945 had no effects on cardiovascular or respiratory systems in the dog and no inhibitory effect on the human ether-a-go-go related gene (hERG) current amplitude recorded from stably transfected human embryonic kidney cells (personal communication).

In adult toxicology studies (inhaled delivery), PC945 was well-tolerated and no safety signals were identified in 14-day studies in rats and dogs. In 13-week studies in rats and dogs, inhalation of PC945 was well-tolerated and there was no systemic toxicity (personal communication). An anticipated dose-related accumulation of drug in alveolar macrophages and an associated inflammatory cell infiltrate were noted, consistent with an overloaded clearance mechanism, and typical of that seen with inhaled medicines [54,55,56].

PC945 is not phototoxic and genetic toxicology tests were all negative. No effect on reproductive performance or foetal development were seen in embryo-foetal development studies in rats and rabbits.

## 4. Clinical Experience

### 4.1. Clinical Trials

The pharmacokinetic and safety profiles of nebulised PC945 from nonclinical and clinical studies in healthy subjects and subjects with mild asthma have been characterised. In nonclinical safety studies, toxicokinetics were initially assessed following daily 2 h inhalation for 14 days. C_max_ occurred 4 h (rats) or immediately (dogs) after a single dose. PC945 lung concentrations were substantially higher (>2000-fold) than those in plasma. The profile of high lung exposure with low plasma concentrations was confirmed in subsequent studies where rats and dogs were dosed with nebulised PC945 for 3 months. In both the 14 day- and 3-month studies, the PC945 concentration data provided clear evidence of accumulation in the lung on repeat dosing, with consequently higher plasma concentrations.

In the First in Human (FIH) study (ClinicalTrials.gov Identifier: NCT02715570) [57,58], clinical safety and pharmacokinetics were assessed following single inhaled emitted (i.e., ex nebuliser) doses of up to 10 mg, 7-day repeat doses (5 mg once daily) in healthy subjects and following a single 5 mg dose in subjects with mild asthma. After a single 5 mg emitted dose, the geometric mean plasma C_max_ was <1 ng/mL in both healthy subjects and subjects with mild asthma 4–5 h after dosing. Following repeat, once daily inhalation (5 mg), day 7 plasma C_max_ was approximately 1 ng/mL 45 minutes after dosing. Increases in C_max_ and AUC_0–24h_ were approximately dose-proportional across the dose range (0.5–10 mg emitted doses). PC945 administration was well tolerated in both healthy subjects and subjects with mild asthma and, importantly for the inhaled route, no clinically significant lung function changes (defined as >15% change from baseline), nor evidence of acute bronchospasm, were observed.

PC945 pharmacokinetic profile translated from nonclinical species to humans, showing slow absorption from lungs and low systemic exposure, thereby limiting the potential for adverse side effects and drug interactions commonly seen with systemically delivered azoles.

### 4.2. Special Needs

PC945 had been supplied to fulfil the special needs of patients with serious or life-threatening *Aspergillus* infections under a Special Needs programme, regulated within the United Kingdom by the Medicines and Healthcare products Regulatory Agency. The outcome of PC945 treatment has been reported in the first two patients who developed invasive pulmonary aspergillosis due to infections with *A. fumigatus* complex susceptible to itraconazole, voriconazole, echinocandins and amphotericin B, shortly following lung transplantation (LT) [59].

**Figure 5:**
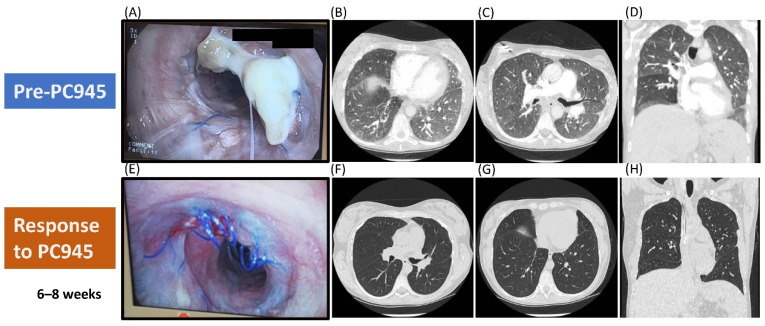
Refractory Invasive Pulmonary Aspergillosis Post Lung Transplant. Pre-exposure images (**A–D**) and response to Nebulised PC945 Treatment (**E–H**). Bronchoscopic images of the anastomosis: (**A**) Refractory infection pre-PC945 treatment, (**E**) After 8 weeks of PC945 treatment. Chest CT scans: (**B–D**) CT angiogram pre-PC945 treatment, (**F–H**) CT scan after 6 weeks of PC945 treatment. Adapted from [59] (and CT scans from Dr P Dalal, personal communication).

The first patient was a 29-year-old woman who developed pneumonia four weeks post-LT for cystic fibrosis. *A. fumigatus* complex was cultured and galactomannan (GM) was strongly positive on bronchoalveolar lavage (BAL). She received caspofungin, isavuconazole, replaced by posaconazole due to gastroenteric intolerance, and nebulised amphotericin B deoxycholate (AmpBD) 25 mg twice daily. The pneumonia improved but the follow-up bronchoscopies revealed a hyphal mass entwined with bronchial anastomotic sutures despite four weeks of antifungal treatment. Terbinafine was added, but the mass continued to increase over the following six weeks (Figure 5A). Biopsy confirmed fungal hyphae infiltrating the cartilage. Due to disease progression, nebulised PC945 was commenced in addition to continued oral posaconazole and terbinafine. Serial bronchoscopy showed improvement after two weeks and complete dissolution of the fungal mass after 8 weeks (Figure 5E). There was complete resolution of ground glass infiltration seen in both lungs on CT after 6 weeks on nebulised PC945 treatment (Figure 5F–H compared versus Figure 5B–D). She received nebulised PC945 for 3 months and remained free from infection three months after completion, whilst continuing posaconazole and terbinafine.

The second patient was a 47-year-old man transplanted for alpha1-antitripsin deficiency. Three months post-LT, a fungal mass with an underlying cavity developed on the left anastomosis. This progressed despite ongoing treatment with caspofungin and nebulised AmpBD. *A. fumigatus* complex was cultured, and the GM was strongly positive in BAL. Oral posaconazole and terbinafine were added, but despite therapeutic plasma levels, there was no improvement after three weeks. Nebulised PC945 was added and posaconazole and terbinafine continued. After two weeks on nebulised PC945, bronchoscopy showed significant shrinkage of the fungal mass and at one month the anastomosis appeared normal with no microbiological evidence of fungal infection.

Plasma levels of PC945 ranged from 2–5 ng/mL in both subjects. The immunosuppressant doses did not need to be adjusted in either patient when nebulised PC945 started and stopped. Nebulised PC945 was well tolerated with no adverse effects in either patient.

No dose adjustments were required in any patient as a result of drug interactions between PC945 and concomitant immunosuppressive or other therapies.

## 5. Conclusions and Future Prospects

*Aspergillus* species are the most important causes of invasive and chronic pulmonary fungal infections. Optimal treatment and prophylaxis of patients at risk of invasive pulmonary aspergillosis is sub-optimal today, being hampered by drug–drug interactions and the narrow therapeutic windows of many currently available antifungal agents, where significant side effects occur at systemic drug concentrations that are often sub-therapeutic at the site of infection in the lung or airway. Treatment success rates for pulmonary *Aspergillus* infections remain disappointingly low, falling well short of the success rates achieved with antibiotics against pulmonary bacterial infections, for example. The need for treatment of *Aspergillus* infections in patients with chronic lung diseases including cystic fibrosis, severe asthma and COPD is currently under diagnosed and under-treated due to the poor risk-benefit of currently available therapies. There is, therefore, a significant unmet need for an antifungal agent that can effectively treat pulmonary aspergillus infections that has a lower risk of significant side effects and drug interactions than approved antifungal agents.

PC945 aqueous suspension, presented as a ready-to-use suspension compatible with commercially available nebulisers, has the potential to address these concerns. As a bespoke, novel inhaled medicine, nebulised suspensions of PC945 have been found to deliver high concentrations in the lung while only producing low systemic exposure.

Evidence that PC945 aqueous suspension has the potential to deliver the intended safety and efficacy profile is now provided by both nonclinical and clinical data. Preclinically, the safety assessment studies in the rat and dog, together with intranasal efficacy studies in the mouse, demonstrated that dosing yields antifungal activity with high concentrations of the compound in the lung but associated with low systemic concentrations. Albeit that the clinical experience to date with nebulised PC945 aqueous suspension is limited, the first-in-human study confirmed a favourable pharmacokinetic profile with low systemic concentrations, long residence time and accumulation in the lung after dosing, coupled with good tolerability and no local irritancy. Finally, the patient case histories obtained to date are very encouraging.

Overall, the results obtained with PC945 thus far appear to validate the notion that using inhaled delivery to achieve high lung concentrations coupled with low systemic concentrations of a novel, bespoke triazole would provide a valuable addition to physicians’ options. The company is now working with regulatory agencies to establish a plan to complete the product’s clinical development.

## Figures and Tables

**Table 1:** Effects of PC945 and known anti-fungal agents against *Aspergillus fumigatus*.

Test Agent	MIC (µg/mL) ^a^		
	Geo Mean ^b^	MIC_50_	MIC_90_
PC945	0.17	0.125	1
Voriconazole	0.42	0.5	1
Posaconazole	0.1	0.063	0.5

^a^ All MICs were determined with EUCAST method. ^b^ Geo-mean = geometric mean. Adapted from [41].

**Table 2:** Summary of studies undertaken which support the investigation of PC945 in clinical studies.

Binding assays in vitro to assess potential for off-target interactions In vitro and in vivo genotoxicology Safety pharmacology (cardiovascular and behavioural) Oral bioavailability and interaction with CYP enzymes 14 day- and 13 week-repeat dose safety assessment (inhaled dosing) in rats and dogs Reproductive toxicology Phototoxicology
